# Preoperative stereotactic radiotherapy to prevent pancreatic fistula in high-risk patients undergoing pancreatoduodenectomy (FIBROPANC): prospective multicentre phase II single-arm trial

**DOI:** 10.1093/bjs/znae327

**Published:** 2025-02-01

**Authors:** Leonoor V Wismans, Tessa E Hendriks, J Annelie Suurmeijer, Joost J Nuyttens, Anna M Bruynzeel, Martijn P Intven, Lydi M van Driel, Roel Haen, Roeland F de Wilde, Bas Groot Koerkamp, Olivier R Busch, Jaap Stoker, Joanne Verheij, Arantza Farina, Onno J de Boer, Michail Doukas, Ignace H de Hingh, Daan J Lips, Erwin van der Harst, Geertjan van Tienhoven, Casper H van Eijck, Marc G Besselink, the Dutch Pancreatic Cancer Group, Annalisa Comandatore

**Affiliations:** Erasmus Medical Center Cancer Institute, Erasmus MC, University Medical Center Rotterdam, Rotterdam, The Netherlands; Department of Surgery, Erasmus MC, University Medical Center Rotterdam, Rotterdam, The Netherlands; Department of Surgery, Amsterdam UMC, location University of Amsterdam, Amsterdam, The Netherlands; Cancer Center Amsterdam, Amsterdam, The Netherlands; Department of Surgery, Leiden University Medical Center, Leiden, The Netherlands; Dutch Institute for Clinical Auditing, Leiden, The Netherlands; Department of Surgery, Amsterdam UMC, location University of Amsterdam, Amsterdam, The Netherlands; Cancer Center Amsterdam, Amsterdam, The Netherlands; Department of Radiation Oncology, Erasmus University Medical Center, Rotterdam, The Netherlands; Department of Surgery, Amsterdam UMC, location University of Amsterdam, Amsterdam, The Netherlands; Department of Radiation Oncology, Amsterdam UMC, location Vrije Universiteit, Amsterdam, The Netherlands; Department of Radiation Oncology, University Medical Center Utrecht, Utrecht, The Netherlands; Department of Gastroenterology and Hepatology, Erasmus University Medical Center, Rotterdam, The Netherlands; Erasmus Medical Center Cancer Institute, Erasmus MC, University Medical Center Rotterdam, Rotterdam, The Netherlands; Department of Surgery, Erasmus MC, University Medical Center Rotterdam, Rotterdam, The Netherlands; Erasmus Medical Center Cancer Institute, Erasmus MC, University Medical Center Rotterdam, Rotterdam, The Netherlands; Department of Surgery, Erasmus MC, University Medical Center Rotterdam, Rotterdam, The Netherlands; Erasmus Medical Center Cancer Institute, Erasmus MC, University Medical Center Rotterdam, Rotterdam, The Netherlands; Department of Surgery, Erasmus MC, University Medical Center Rotterdam, Rotterdam, The Netherlands; Department of Surgery, Amsterdam UMC, location University of Amsterdam, Amsterdam, The Netherlands; Cancer Center Amsterdam, Amsterdam, The Netherlands; Cancer Center Amsterdam, Amsterdam, The Netherlands; Department of Radiology and Nuclear Medicine, Amsterdam UMC, location University of Amsterdam, Amsterdam, The Netherlands; Cancer Center Amsterdam, Amsterdam, The Netherlands; Department of Pathology, Amsterdam UMC, location University of Amsterdam, Amsterdam, The Netherlands; Cancer Center Amsterdam, Amsterdam, The Netherlands; Department of Pathology, Amsterdam UMC, location University of Amsterdam, Amsterdam, The Netherlands; Cancer Center Amsterdam, Amsterdam, The Netherlands; Department of Pathology, Amsterdam UMC, location University of Amsterdam, Amsterdam, The Netherlands; Erasmus Medical Center Cancer Institute, Erasmus MC, University Medical Center Rotterdam, Rotterdam, The Netherlands; Department of Pathology, Erasmus University Medical Center, Rotterdam, The Netherlands; Department of Surgery, Catharina Hospital, Catharina Cancer Institute, Eindhoven, The Netherlands; Department of Surgery, Medisch Spectrum Twente, Enschede, The Netherlands; Department of Surgery, Maasstad Hospital, Rotterdam, The Netherlands; Department of Surgery, Amsterdam UMC, location University of Amsterdam, Amsterdam, The Netherlands; Department of Radiation Oncology, Amsterdam UMC, location University of Amsterdam, Amsterdam, The Netherlands; Erasmus Medical Center Cancer Institute, Erasmus MC, University Medical Center Rotterdam, Rotterdam, The Netherlands; Department of Surgery, Erasmus MC, University Medical Center Rotterdam, Rotterdam, The Netherlands; Department of Surgery, Amsterdam UMC, location University of Amsterdam, Amsterdam, The Netherlands; Cancer Center Amsterdam, Amsterdam, The Netherlands

## Abstract

**Background:**

Postoperative pancreatic fistula is the main driver of morbidity and mortality after pancreatoduodenectomy. In high-risk patients, the rate of postoperative pancreatic fistula approaches 50%, whereas it is below 5% in patients with pancreatic cancer who receive neoadjuvant chemoradiotherapy. The aim of this study was to evaluate the safety, feasibility, and efficacy of preoperative stereotactic body radiotherapy on the pancreatic neck transection margin in high-risk patients undergoing pancreatoduodenectomy to prevent postoperative pancreatic fistula.

**Methods:**

In this prospective multicentre open-label single-arm trial (progressing from a safety run-in phase to a phase II design), patients undergoing pancreatoduodenectomy for neoplasms other than pancreatic ductal adenocarcinoma received a single preoperative stereotactic body radiotherapy dose of 12 Gy. Primary endpoints included safety (less than or equal to 15% grade 3–5 toxicity), feasibility (a significant change in pancreatic texture measured using a durometer), and efficacy (a 15% reduction in the grade B/C postoperative pancreatic fistula rate compared with patients from the Dutch Pancreatic Cancer Audit who were eligible but not included in this study). Secondary endpoints assessed tissue fibrosis (collagen density).

**Results:**

Overall, 38 patients were included, of whom 33 (87%) completed the study protocol and were included in the per-protocol analysis. The safety cut-off was met, with 3% grade 3–5 toxicity. Pancreatic tissue treated with stereotactic body radiotherapy showed increased firmness using a durometer (median of 47 (interquartile range 36–57) *versus* 37 (interquartile range 30–41) Shore OO units; *P* < 0.001) and a higher collagen density (median of 6.1% (interquartile range 4.4%–9.5%) *versus* 4.6% (interquartile range 2.5%–7.4%); *P* = 0.003). The grade B/C postoperative pancreatic fistula rate with stereotactic body radiotherapy was 57.6% (95% c.i. 41% to 74%), compared with 34% (95% c.i. 27% to 42%) in audit controls (*P* = 0.011).

**Conclusion:**

Preoperative stereotactic body radiotherapy is safe in high-risk patients undergoing pancreatoduodenectomy and increases parenchymal firmness and fibrosis, but fails to show evidence of efficacy.

## Teaser

The FIBROPANC study investigated the safety, feasibility, and efficacy of preoperative stereotactic radiotherapy on the pancreatic neck transection margin to reduce postoperative pancreatic fistula in high-risk patients undergoing pancreatoduodenectomy. A single fraction of high-dose stereotactic body radiotherapy increased parenchymal firmness and fibrosis, but did not reduce the postoperative pancreatic fistula rate.

## Introduction

Postoperative pancreatic fistula (POPF) remains the primary driver of morbidity and mortality after pancreatoduodenectomy^1–3^. The healthcare costs of one episode of POPF amount to 20,000–50,000 US dollars^4,5^. Despite numerous trials attempting to optimize surgical techniques, timely percutaneous drainage, and pharmacological interventions, the risk and severity of POPF after pancreatoduodenectomy did not decline^6,7^. Therefore, there is an urgent need for effective perioperative interventions to reduce the risk of POPF, especially in high-risk patients.

In recent studies, preoperative chemoradiotherapy was associated with a reduced rate of POPF in patients with pancreatic cancer^8–10^. The suggested protective effect of radiotherapy is likely mediated through the induction of fibrosis^11,12^. Radiation-induced fibrosis could increase parenchymal firmness and prevent tearing by increasing the suture hold capacity. However, there are no oncological indications for preoperative chemoradiotherapy in patients undergoing pancreatoduodenectomy for indications other than pancreatic cancer.

Patients with diagnoses other than pancreatic cancer requiring pancreatoduodenectomy have a much higher risk of developing POPF^13,14^. Prominent risk factors for POPF include a soft pancreatic parenchyma and a small main pancreatic duct (MPD) diameter of less than or equal to 3 mm, classified as ‘grade D’ by the International Study Group for Pancreatic Surgery (ISGPS)^15,16^. The POPF risk in this patient category increases to over 30%^16^. Yet, prospective studies using preoperative radiotherapy to reduce the rate of POPF in these high-risk patients are lacking.

Stereotactic body radiotherapy (SBRT) offers precise targeting and concentrated high doses of radiation. The authors hypothesize that a single fraction of SBRT targeted at a 4 cm area centred around the future transection site of the pancreas (that is the neck) may cause local fibrosis, resulting in firmer pancreatic tissue, which might reduce the risk of POPF. To evaluate radiotherapy-induced changes in pancreatic tissue and their impact on the development of POPF, standardized methods to quantify firmness and fibrosis are needed. However, the assessment of pancreatic firmness relies solely on a surgeon’s subjective evaluation, as methods to assess pancreatic texture are lacking^17–19^.

The aim of this study was to investigate the safety, feasibility, and efficacy of single-fraction SBRT targeted at the future pancreatic transection site to reduce the risk of POPF in patients at high risk undergoing pancreatoduodenectomy for (pre-)malignant tumours other than pancreatic cancer, including objective measurement of pancreatic texture and fibrosis.

## Methods

FIBROPANC was a prospective multicentre open-label single-arm trial (progressing from a safety run-in phase to a phase II design). Patients scheduled for pancreatoduodenectomy at high risk of developing POPF received preoperative, single-fraction SBRT at the future pancreatic neck transection site. The study protocol has been published previously^20^. The initial phase aimed to assess safety and feasibility; if safety and feasibility criteria were met, additional patients were enrolled to evaluate the intervention’s efficacy in reducing grade B/C POPF.

### Study population

Eligible patients included those scheduled for pancreatoduodenectomy for a (pre-)malignant tumour other than pancreatic ductal adenocarcinoma, with an MPD diameter of less than or equal to 3 mm. Additional inclusion criteria were a WHO performance status of less than or equal to grade 2, age greater than or equal to 18 years, and suitability to undergo both SBRT and pancreatoduodenectomy. Exclusion criteria included patients undergoing pancreatoduodenectomy for (suspected) pancreatic ductal adenocarcinoma, chronic pancreatitis, or benign neoplasms. The study ran between March 2021 and July 2023.

### Study intervention and protocol completion

**Fig. 1:**
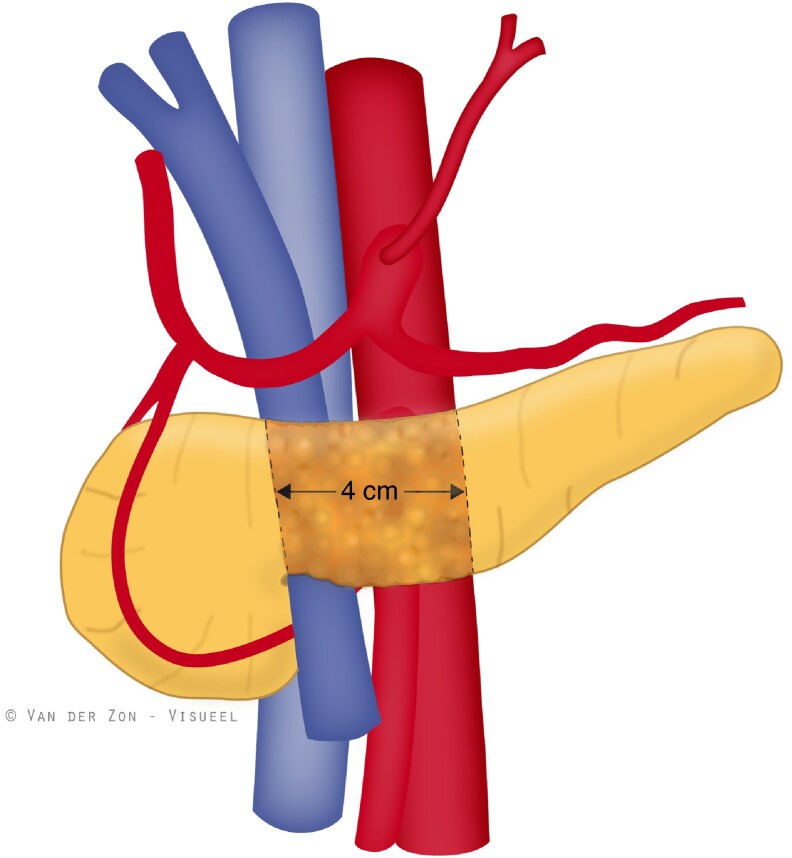
Gross target volume for stereotactic body radiotherapy: from the right-sided border of the portal vein 4 cm towards the spleen

The SBRT was delivered in a single fraction of 12 Gy. The dosage was calculated as described previously^20^ and was delivered using MRI-guided SBRT or CyberKnife. CyberKnife was performed using fiducial markers in the pancreas (endoscopic ultrasound (EUS)-guided placement), plastic fiducial clips in the duodenum (endoscopic placement), or CT-guided internal target volume (non-invasive). MRI-guided SBRT was a non-invasive procedure. The gross target volume (GTV) included the middle of the pancreatic corpus, covering the intended location of the pancreatic-enteric anastomosis—4 cm towards the spleen from the right-sided border of the portal vein (*Fig. 1*). The study protocol describes additional target volumes, treatment planning, and dose prescription information^20^. Surgery was scheduled at least 4 weeks after SBRT to allow fibrosis to develop; if possible, an interval of 6 weeks was maintained in the case of premalignant disease. Protocol completion was defined as the ability to receive SBRT and maintain a post-SBRT interval of 4 weeks without causing any additional delay of the scheduled pancreatoduodenectomy.

### Primary outcomes

#### Safety

For safety, all toxicities of grade 3–5 potentially related to the preparation or administration of SBRT in all included patients (intention-to-treat analysis) were considered events. All toxicities occurring up to surgery or at least 21 days after SBRT administration were collected. The potential relation to SBRT of all intraoperative and postoperative complications occurring within 30 days after surgery was evaluated to ensure that indirect toxicities of the study treatment were also documented. Safety was reached if less than or equal to 15% of patients experienced grade 3–5 study intervention-related toxicity.

#### Feasibility

The intervention’s proof of principle was represented by the feasibility, defined as a significant change in pancreatic texture between the resection site (the irradiated tissue) and the uncinate process (the non-irradiated tissue) within the same patient using a durometer (Model DD-4 Digital Durometer Type Shore OO, Rex Gauge Company, Lake Zurich, 11, USA).

#### Efficacy

Efficacy was defined as a grade B/C POPF risk reduction of 15% from the population risk in audit controls. Data from the Dutch Pancreatic Cancer Audit (DPCA) was used to calculate a population risk for patients that met the study inclusion criteria during the study interval (that is MPD less than or equal to 3 mm and a diagnosis other than pancreatic ductal adenocarcinoma operated on within the participating study centres, during the study interval). Before the study interval (2014–2018), the predefined population risk was considered greater than 25%, as described in the study protocol^20^.

### Secondary outcomes

#### Assessment of firmness, fibrosis, and pancreatitis

Several techniques were implemented to evaluate the SBRT-induced effect on pancreatic tissue. First, intraoperatively, the surgeon assessed whether palpable local fibrosis was present and assessed any visual impact of SBRT at the pancreatic neck transection site (the irradiated field) compared with the uncinate process (the non-irradiated field). Secondly, a sample from the resection site (an irradiated sample) and a sample from the uncinate process (a non-irradiated sample) were taken during the processing of the specimen, as described in the study protocol^20^. The pathologist examined both samples for fibrosis macroscopically.

Third, the durometer was used to quantify the effect of radiotherapy on pancreatic texture. The correlation between durometric measurement and surgeon evaluation was demonstrated in previous studies^18,19,21^. Durometric measurement was standardized using an operating stand to optimize validity and minimize inter-observer bias (Figure S1). Durometer values were measured in Shore OO units, ranging from 0 (soft) to 100 (firm). The mean result of three repeated measurements per sample at the resection site (the irradiated tissue) and the uncinate process (the non-irradiated tissue) within the same patient was documented. The standard operating procedure for sample collection and durometric measurement can be found in the study protocol^20^. Fourth, histopathological evaluation using Picrosirius red staining was conducted objectively to quantify collagen, the main component of fibrosis. Slides from both samples were stained to identify post-radiation differences in collagen content. Histology slides were digitised using a Philips Ultrafast Scanner (Philips Digital Pathology Systems, Best, the Netherlands) at 40x resolution (0.25 microns/pixel). All slide images were converted to BigTiff, and the total tissue area and the amount of Picrosirius red staining were quantified using QuPath v0.5.1 and expressed as a percentage of positive staining.^22^

Fifth, Haematoxylin and eosin (H & E)-stained slides were assessed, focusing on histopathological features indicative of pancreatitis in the irradiated sample compared with the non-irradiated sample. Additionally, durometric measurement and picrosirius red staining were performed for control patients. Control samples were collected from eligible patients who did not receive the study intervention.

#### Additional outcomes

Other outcomes included major complications, pancreatic surgery-related complications up to 30 days after surgery or during hospital stay, duration of hospital stay, and readmission rate within 30 days after discharge. Pancreatic surgery-related complications included POPF^2^, post-pancreatectomy haemorrhage (PPH)^23^, chyle leakage^24^, bile leakage^25^, and delayed gastric emptying (DGE)^26^, all defined as grade B/C according to the ISGPS or International Study Group of Liver Surgery (ISGLS) criteria. Mortality included in-hospital/30-day mortality rates (that is in-hospital mortality during primary admission) and 90-day mortality rates. Postoperative measurements of HbA1c for new-onset diabetes mellitus and faecal elastase levels for exocrine pancreatic function were in accordance with institutional standards.

### Ethical approval, data, and sample collection

This study was performed in line with the principles of the Declaration of Helsinki^27^. The Dutch Pancreatic Cancer Group (DPCG) supported the study. The institutional review boards of both participating centres approved the study protocol and amendments (Amsterdam UMC MEC 2020-079 and Erasmus MC MEC 2020-0688). The trial was registered on the Central Committee on Research Involving Human Subjects Register with trial number NL72913 and on ClinicalTrials.gov with trial number NCT05641233. Biomaterials of control patients were included in the biobank (Amsterdam UMC MEC 2014-094 and Erasmus MC MEC 2015-085). All patients provided written informed consent. Study baseline characteristics included sex, age during surgery, BMI, WHO performance status, ASA grade, Charlson co-morbidity index, and postoperative histopathological diagnosis. Treatment characteristics included SBRT type, marker placement, interval between SBRT and surgery, surgical approach, and MPD diameter at the pancreatic neck. Perioperative characteristics included use of somatostatin analogues, intraoperative drain placement, and pancreatic texture.

### Sample size calculation

Of the 20 patients eligible for the safety run-in phase, it was considered acceptable for up to 3 patients (less than or equal to 15%) to experience grade 3–5 toxicity related to the intervention, based on an expected 15% reduction in POPF. For feasibility, a one-sample *t* test with a 5% one-sided significance level had 80% power to detect the difference between a null hypothesis mean of 11 and an alternative mean of 25 (using a durometer), assuming that the standard deviation was 21 based on previous literature^19,20^. For the second phase, focusing on clinical efficacy, an additional 13 patients were needed to calculate a reduction of 15% in POPF in 33 patients (α 0.05, power 0.80), with an intended 15% reduction. Flemming’s procedure was used for calculation. Complete safety, feasibility, and POPF measurements were required in 33 patients who completed the study protocol to prevent type II errors. Patients were replaced if an endpoint could not be evaluated (for example patients did not undergo resection due to the discovery of metastases during exploratory laparotomy or withdrawal of informed consent before the intervention) or if they underwent surgery within 4 weeks after SBRT.

### Statistical analysis

Continuous data are presented as median (interquartile range (i.q.r.)) and categorical data are presented as *n* (%). Differences in durometer outcomes and picrosirius red density were determined using Wilcoxon matched-pairs signed rank tests. For statistical analysis, *P* < 0.050 was deemed significant. All statistical tests were two-tailed and analysis was performed using R statistical software (version 4.1.1). Figures were made using GraphPad Prism software (version 8.0).

## Results

**Fig. 2:**
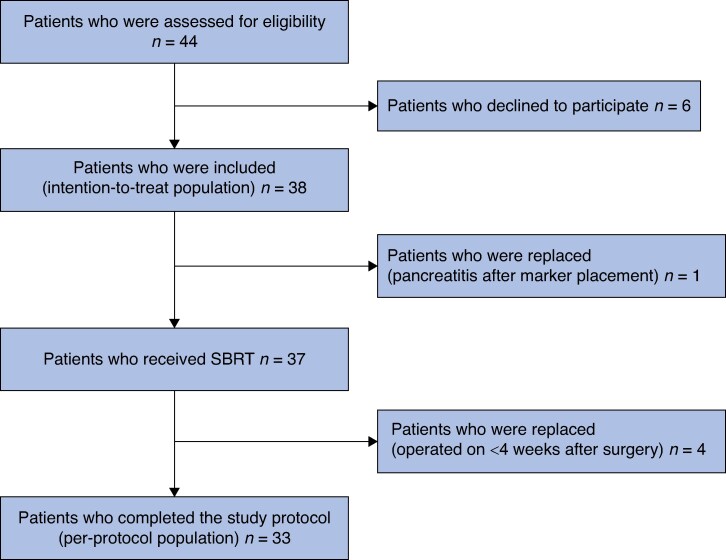
Study flow chart SBRT, stereotactic body radiotherapy.

This phase II study included 38 patients from two centres after the safety run-in phase demonstrated the intervention’s safety and feasibility. Of the 38 patients who were included, 33 (86.8%) completed the study protocol and were included in the per-protocol analysis. In four patients (10.5%), the time between SBRT and surgery was less than 4 weeks and one patient (2.6%) did not receive SBRT due to post-EUS-guided fiducial marker placement-induced pancreatitis. The study flow chart is shown in *Fig. 2*. The median age of the patients was 69 (i.q.r. 61–72) years and 24 patients (63.2%) were men. Most patients (63%) had an ASA grade between 0–II and the most common postoperative histopathological diagnoses were cholangiocarcinoma (9 patients, 23.7%) and duodenal adenocarcinoma (9 patients, 23.7%) (*Table 1*).

**Table 1:** Baseline characteristics

	All patients (*n* = 38)	Per-protocol (*n* = 33)
**Sex**		
Male	24 (63.2)	21 (63.6)
Female	14 (36.8)	12 (36.4)
Age (years), median (i.q.r.)	69 (61–72)	69 (66–72)
BMI >25 kg/m^2^	15 (39.5)	11 (33.3)
**WHO performance status**		
Fully independent	8 (21.1)	8 (24.2)
Partially independent	30 (78.9)	30 (75.8)
Fully dependent	0 (0.0)	0 (0.0)
**ASA grade**		
0–II	24 (63)	28 (84.8)
III–IV	14 (37)	13 (39.4)
**Charlson co-morbidity index**		
0–1	33 (86.8)	28 (84.8)
≥2	5 (13.2)	5 (15.2)
**Postoperative pathology**		
Cholangiocarcinoma	9 (23.7)	8 (24.2)
Duodenal adenocarcinoma	9 (23.7)	7 (21.2)
Papillary cancer	7 (18.4)	5 (15.2)
Neuroendocrine neoplasm	5 (13.2)	5 (15.2)
IPMN, SPN, or MCN	2 (5.3)	2 (6.1)
Pancreatic adenocarcinoma	1 (2.6)	1 (3.0)
Other*	5 (13.2)	5 (13.2)

Values are *n* (%) unless otherwise indicated. *Papillary adenoma, low-grade dysplasia, and no disease. i.q.r., interquartile range; IPMN, intraductal papillary mucinous neoplasm; SPN, solid pseudopapillary neoplasm, MCN, mucinous cystic neoplasm.

### Study intervention

Of the 38 patients who were included, 37 (97.3%) received a single dose of 12 Gy SBRT before surgery using MRI-guided SBRT (16 patients, 42.1%) or CyberKnife (21 patients, 55.3%). CyberKnife was performed using fiducial markers in six patients (16%)—EUS-guided in the pancreas (5 patients) or endoscopically in the duodenum (1 patient). Most patients (32 patients, 84%) were treated using MRI-guided SBRT or CyberKnife without fiducial markers. The median time from the date of surgical indication to SBRT was 15 (i.q.r. 7–22) days. In the per-protocol population, the median interval between SBRT and surgery was 5.8 (i.q.r. 4.6–6.4) weeks (*Table 2* and *Table 3*).

**Table 2:** Treatment and perioperative characteristics

	All patients (*n* = 38)	Per-protocol (*n* = 33)
**Type of SBRT**		
MRI-guided	16 (42.1)	15 (45.5)
CyberKnife	21 (55.3)	18 (54.5)
No SBRT	1 (2.6)	0 (0.0)
**Fiducial marker placement**		
EUS-guided	5 (13.2)	4 (12.1)
Endoscopic fiducial clip placement	1 (2.6)	1 (3.0)
No fiducial marker placement	32 (84.2)	28 (84.8)
Interval between inclusion and SBRT (days), median (i.q.r.)	15 (7–22)	15 (7–22)
Interval between SBRT and surgery (weeks), median (i.q.r.)	5.4 (4.4–6.4)	5.8 (4.6–6.4)
**Surgical approach to pancreatoduodenectomy**		
Open	14 (36.8)	12 (36.4)
Robot-assisted	22 (57.9)	21 (63.6)
Diameter of the main pancreatic duct at the pancreatic neck (mm), median (i.q.r.)	3.0 (2.0–3.0)	3.0 (2.0–3.0)
Use of somatostatin analogues	21 (62)	20 (60.6)
Intraoperative drain placement	21 (55.2)	20 (60.6)
Intraoperative blood loss (ml), median (i.q.r.)	400 (210–700)	300 (175–600)
Intraoperative time (min), median (i.q.r.)	352 (245–467)	354 (278–444)
**Pancreatic texture**		
Soft/normal	34 (89.5)	30 (90.9)
Firm	4 (10.5)	3 (9.1)
Surgeon assessment, local firmness*		
No	32 (84.8)	28 (84.8)
Yes, more firm	6 (15.6)	5 (15.1)
Surgeon assessment, local visual effects*		
No effects	32 (84.2)	27 (81.8)
Oedema	1 (2.6)	1 (3.0)
Thickening of the main pancreatic duct	5 (13.2)	4 (12.1)
**Pathological assessment, macroscopic fibrosis†**		
No	24 (63.2)	24 (72.7)
Fibrosis, mild	9 (23.7)	9 (27.2)
Missing	5 (13.2)	0 (0.0)
**Pathological assessment, features of pancreatitis or other inflammatory changes†**		
No	25 (65.8)	25 (75.8)
Yes	4 (10.5)	4 (12.1)
Missing	9 (23.7)	4 (12.1)

Values are *n* (%) unless otherwise indicated. *Comparison between the pancreatic neck transection site (the stereotactic body radiotherapy target area) and the uncinate process. †Comparison between the sample from the resection margin (the irradiated sample) and the sample from the uncinate process (the non-irradiated sample). SBRT, stereotactic body radiotherapy; EUS, endoscopic ultrasound; i.q.r., interquartile range.

**Table 3:** Clinical outcomes

	All patients (*n* = 38)	Per-protocol (*n* = 33)
Grade B/C POPF	22 (57.9)	19 (57.6)
Duration of drainage (days), median (i.q.r.)	34 (17–53)	34 (19–52)
Duration of hospital stay (days), median (i.q.r.)	8 (14–26)	8 (14–23)
Reoperation	4 (10.5)	3 (9)
Grade B/C DGE	13 (34.2)	12 (36.4)
Grade B/C PPH	4 (10.5)	4 (12)
Grade B/C BL	4 (10.5)	3 (9.1)
Pancreatitis	1 (2.6)	1 (3.0)
Readmission	8 (21.2)	8 (24.2)
30-day/in-hospital mortality	1 (2.6)	1 (3)
90-day mortality	1 (2.6)	1 (3)
HbA1C (mmol/mol), median	47.1	47.8
Missing, *n*	18	18
New-onset diabetes	5 (13.2)	5 (15.2)
**Pancreatic insufficiency, faecal elastase measurement, *n***		
No (>200 μg/g)	0	0
Mild–moderate (100–200 μg/g)	0	0
Severe (<100 μg/g)	10	8
Missing	28	25

POPF, postoperative pancreatic fistula; i.q.r., interquartile range; DGE, delayed gastric emptying; PPH, post-pancreatectomy haemorrhage; BL, bile leakage.

### Adverse events related to the study intervention

The safety cut-off of less than or equal to 15% was met; 1 (2.6%) of the 38 included patients experienced grade 3–5 toxicity related to the intervention. This patient, as mentioned earlier, developed grade 3 toxicity because of acute pancreatitis after EUS-guided fiducial marker placement (*Table S1*). No other adverse events occurred after fiducial marker placement. After SBRT, ten patients (27%) experienced grade 1 complaints, such as fatigue (2 patients), nausea (2 patients), and abdominal pain (3 patients). These were self-limiting events without the need for medical intervention. One patient (2%) experienced grade 2 abdominal pain and required pain medication (*Table S2*). Another patient (2%) was diagnosed with asymptomatic colitis using preoperative CT, for which a hemicolectomy was indicated. The latter serious adverse event was deemed to be unrelated to SBRT, as the inflamed area was outside of the irradiated field. In one patient, the postoperative pathology revealed pancreatic ductal adenocarcinoma and the patient started chemotherapy directly after surgery.

### Effect of stereotactic body radiotherapy on firmness and fibrosis

**Fig. 3:**
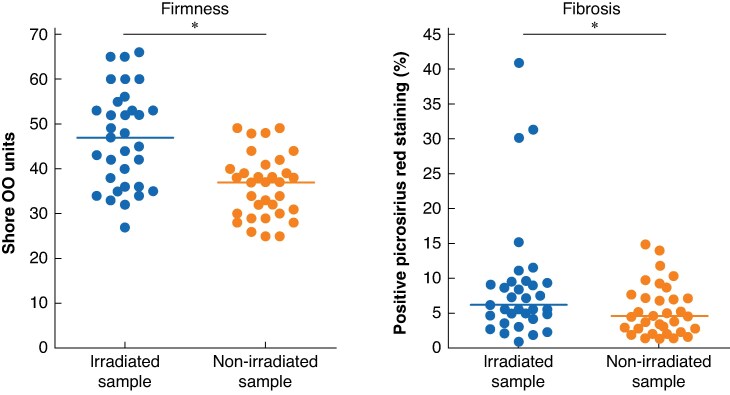
Outcomes of assessments of firmness (measured using a durometer) and fibrosis (collagen density; measured using picrosirius red staining) of the resection margin (the irradiated sample) and the uncinate process (the non-irradiated sample) An asterisk indicates statistical significance (*P* < 0.050).

Feasibility was met; in the 33 patients who completed the study protocol, the pancreatic tissue at the irradiated resection site was significantly more firm, according to durometric measurement, compared with the non-irradiated tissue at the uncinate process (median of 47 (i.q.r. 36–57) *versus* 37 (i.q.r. 30–41) Shore OO units; *P* < 0.001) (*Fig. 3* and *Table S3*). Shore OO units range from 0 (soft) to 100 (firm). The macroscopic assessment of the surgeon and pathologist could not distinguish between irradiated and non-irradiated tissue. In 28 patients (84.9%), as subjectively assessed by the surgeon, SBRT did not induce local firmness at the irradiated field; however, in 5 patients (15.1%), increased firmness was considered present. The pathologist assessed that, in 24 patients (72.2%), fibrosis was not present in the sample of the irradiated tissue. Microscopic assessment showed that the density of the collagen marker picrosirius red was significantly higher in the irradiated sample compared with the non-irradiated sample (median density of 6.1% (i.q.r. 4.4%–9.5%) *versus* 4.6% (i.q.r. 2.5%–7.4%); *P* = 0.003) (*Fig. 3* and *Table S3*). In four patients (12.1%), a small focus of pancreatitis was found in the irradiated sample, with the absence of pancreatitis in the non-irradiated sample (*Table 2*).

Durometric measurement and picrosirius red staining were also performed on collected samples of control patients (that is eligible patients who did not undergo SBRT). Durometric measurement in this control group revealed no significant difference in firmness between the resection site and the uncinate process (7 control patients; median of 40 (i.q.r. 35–56) *versus* 43 (i.q.r. 34–49) Shore OO units respectively; *P* = 0.58); likewise, the density of collagen was similar (3 control patients; median density of 12.2% (i.q.r. 8.1%–28.6%) *versus* 12.1% (i.q.r. 9.0%–16.1%) respectively; *P* = 0.75) (*Table S3*).

### Clinical outcomes

In the per-protocol analysis of 33 patients, 19 patients (57.6% (95% c.i. 41% to 74%)) developed a grade B/C POPF. One of these patients (3.0%) died of sepsis due to multiple intra-abdominal abscesses and bleeding of a pseudoaneurysm of the common hepatic artery. All patients with a grade B/C POPF underwent radiological percutaneous drainage with a median duration of drainage of 34 (i.q.r. 19–52) days. PPH occurred in three patients (9.1%) with a grade B/C POPF and one patient (3.0%) without a grade B/C POPF. Among all patients included in the per-protocol analysis, the median duration of hospital stay was 8 (i.q.r. 14–23) days, and, in three patients (9%), a reoperation was performed (*Table 3*).

### Risk of postoperative pancreatic fistula in audit controls

The risk of a grade B/C POPF in audit controls, that is eligible non-study patients, during the study interval, was 34% (45 of 134 audit controls; 95% c.i. 27% to 42%), compared with 57.6% (95% c.i. 41% to 74%) in the study population (*P* = 0.011). Potential risk factors for POPF in audit controls are shown in *Table S4*.

### Exocrine and endocrine pancreatic function

After pancreatoduodenectomy, 5 of 38 patients (13.2%) were diagnosed with new-onset diabetes and 31 of 38 patients (81.5%) received pancreatic enzyme replacement therapy. Faecal elastase was measured in 10 of 38 patients (26.3%), who all exhibited severe pancreatic insufficiency.

## Discussion

This prospective multicentre open-label phase II single-arm trial demonstrates that a single fraction of 12 Gy SBRT at the future pancreatic neck transection site in patients undergoing high-risk pancreatoduodenectomy is safe, is feasible (as it increases firmness, according to durometric measurement), and induces pancreatic fibrosis. However, no efficacy could be demonstrated, as SBRT appears ineffective in reducing the rate of grade B/C POPF after pancreatoduodenectomy compared with audit controls.

These results cannot be compared with other studies, as this is the first study to assess SBRT with regard to preventing POPF after pancreatoduodenectomy in patients with diagnoses other than pancreatic ductal adenocarcinoma. In the study population, 19 patients (57.6% (95% c.i. 41% to 74%)) developed a grade B/C POPF. During the study interval, the population risk in audit controls was 34% (95% c.i. 27% to 42%). Therefore, the authors cannot exclude the possibility that the intervention induced changes that increased the risk of POPF. The association between POPF and radiotherapy has been predominantly noted in patients with pancreatic ductal adenocarcinoma in the pancreatic head, where obstruction of the MPD leads to pancreatic inflammation and fibrosis^28^. The present study included patients undergoing pancreatoduodenectomy with ‘healthy’ pancreatic tissue. In these patients, a single fraction of SBRT may have caused changes that rendered the tissue more fragile or prone to anastomotic dehiscence. However, surgeons reported no detectable changes in the irradiated tissue in most patients (84.8%) and inflammatory alterations at the resection margin were only observed in four patients (10.5%), without reported difficulties in pancreatic dissection. Without a control group, it is difficult to determine whether the increased POPF rate is directly attributable to SBRT or reflects other patient- or procedure-related factors. Notably, similar to the high POPF rate observed in the present study, a recent prospective study involving 566 high-risk patients reported a POPF rate of 39% after pancreatoduodenectomy^29^. This highlights the significant risk of POPF within this population and underscores the need for effective interventions.

The study intervention resulted in minimal and mild side effects, achieving the safety outcomes. However, one patient, diagnosed with an ampullary adenoma, developed pancreatitis post-EUS-guided fiducial marker placement. Consequently, the patient did not receive SBRT but ultimately underwent pancreatoduodenectomy after recovery from pancreatitis. The potential harm of the study intervention includes impaired exocrine or endocrine pancreatic function. However, the authors do not expect a single fraction of SBRT on a small part of the pancreas to have further impaired endocrine/exocrine function beyond the impact of pancreatoduodenectomy. New-onset diabetes was diagnosed in 13.2% of the patients after pancreatoduodenectomy, which is comparable to the pooled estimate of 16% reported in a recent meta-analysis^30^. Concerning pancreatic function, up to 74% (range 36%–100%) of patients develop exocrine insufficiency after pancreatic surgery, necessitating enzyme replacement therapy^31^, which is similar to the 81.5% of patients in the present study.

As mentioned, FIBROPANC is the first trial to investigate the potential of a single SBRT fraction of 12 Gy to reduce the risk of postoperative complications in high-risk patients. Over the past years, compelling evidence has demonstrated that preoperative chemoradiotherapy reduces the rate of POPF after pancreatoduodenectomy in patients with pancreatic ductal adenocarcinoma^8–10^. PREOPANC was the first RCT to report lower rates of POPF in patients with pancreatic cancer after preoperative chemoradiotherapy compared with immediate surgery (0.0% *versus* 9.2%; *P* = 0.011)^9^. Although preoperative chemoradiotherapy is associated with a reduced rate of POPF, a single fraction of SBRT in the present study appeared ineffective in this high-risk study population. The increased firmness (measured using a durometer) and fibrosis was insufficient to reduce the rate of POPF. Several factors could potentially explain this. A time–effect relationship between the post-radiation interval and pancreatic fibrosis may be present. The late fibroproliferative phase, characterized by tissue reorganization and accumulation of extracellular matrix proteins, typically occurs after several months^32^. For this reason, further postponing surgery could be beneficial, as this allows the pancreatic tissue more time to modulate. However, in the present study, in patients with an interval between SBRT and surgery of greater than 6 weeks, the rate of grade B/C POPF was 53%. Further, a single fraction of 12 Gy (Equivalent Dose in 2 Gray Fractions [EQD2Gy] = 36 Gy, α/β = 3 Gy) monotherapy may not be sufficient to induce the fibrosis necessary to reduce the risk of POPF. In previous studies, radiotherapy was combined with chemotherapy, working as a radiosensitizer, and was provided in multiple fractions. The current dose of 12 Gy was calculated based on the PREOPANC trial (15 × 2.4 Gy, EQD2Gy∼38 Gy), but was reduced to a single fraction to minimize patient burden and time delay to operation^9^. The authors presumed that the late effect rather than the acute effect of radiotherapy was responsible for the accumulation of fibrosis. Therefore, the regimen of 1 × 12 Gy corresponded to a biological equivalent dose for the late effect in the PREOPANC trial, but to a lower biological equivalent dose of 26 Gy_10_ for acute effects. The latter may contribute to the observed inefficacy of the current regimen.

The association between preoperative chemoradiotherapy and the rate of POPF may be explained by the prolonged time to surgery, rather than the chemoradiotherapy itself. In most patients with a pancreatic ductal adenocarcinoma located in the head, the interval of MDP obstruction is prolonged by postponing the surgery in the preoperative setting, which could result in a firmer pancreatic texture^28^. The randomized PREOPANC-2 trial (neoadjuvant chemoradiotherapy *versus* neoadjuvant chemotherapy) and randomized PREOPANC-3 trial (immediate surgery *versus* perioperative chemotherapy) expect to provide definite answers on the impact of preoperative therapy on POPF^33,34^.

The population risk of POPF before the start of the study (2014–2018) was 27.5% and increased to 35.6% (47 of 134, 95% c.i. 32% to 40%) during the study interval^20^. The increase in the rate of POPF during the study interval, both in the audit controls and in the study patients, is partly explained by the implementation of the nationwide PORSCH trial^6^. This trial recommended radiological drainage at a low threshold, which, by definition, results in a grade B POPF.

As pancreatic texture is the most critical risk factor for POPF, multiple studies have investigated methods to assess pancreatic texture^17–19^. It has been reported that using a durometer allows a surgeon to successfully distinguish between a non-pathological soft pancreas and a hard pancreas^19^. FIBROPANC is the first trial to objectively assess radiation-induced firmness using a durometer in a standardized way and radiation-induced fibrosis by computational collagen quantification using picrosirius red staining. The durometer and collagen quantification using picrosirius red staining discriminated between irradiated and non-irradiated tissue, whereas the surgeon did not observe these differences. The absence of differences in firmness and fibrosis between the resection site and the uncinate process in control patients increased the validity of these measurements. Therefore, using a durometer using a standardized method and collagen quantification using picrosirius red staining is valid for assessing firmness and pancreatic fibrosis in future clinical trials.

The findings of this study should be interpreted considering limitations. Due to the small sample size and single-arm non-randomized design, drawing definitive conclusions regarding the potential positive or negative impact of the intervention on POPF is challenging. Based on the observed POPF rate, the authors anticipate that a randomized trial will not be able to demonstrate the efficacy of SBRT in this population. It may be worthwhile to explore whether extending the time frame between SBRT and surgery might allow for more fibrosis to accumulate, as discussed previously. Also, administering a higher biological equivalent dose for acute effects (for example 3 fractions of 8 Gy each) may induce fibrosis more extensively. Exposing patients to the potential risks associated with these interventions could be justified based on the outcomes of the present trial. Total pancreatectomy has been proposed as an alternative to high-risk pancreatoduodenectomy to avoid POPF^29,35^. As total pancreatectomy results in complete endocrine and exocrine insufficiency, this approach should only be considered as a last resort. Further trials should focus on combining effective interventions to prevent POPF in a multicentre RCT.

## Collaborators

The FIBROPANC trial was conducted on behalf of the Dutch Pancreatic Cancer Group. Other collaborator: Annalisa Comandatore (General Surgery Unit, Cisanello Hospital, Department of Translational Research and New Technologies in Medicine and Surgery, University of Pisa, Pisa, Italy).

## Supplementary Material

znae327_Supplementary_Data

## Data Availability

Data are available from the corresponding author upon reasonable request.
