# Supplementary material for: Preoperative stereotactic radiotherapy to prevent pancreatic fistula in high-risk patients undergoing pancreatoduodenectomy (FIBROPANC): prospective multicentre phase II single-arm trial
Source: Br J Surg. 2025 Feb 1;112(2):znae327. doi: 10.1093/bjs/znae327 (PMC11785728; doi:10.1093/bjs/znae327)
Supplement: znae327_Supplementary_Data [file znae327_supplementary_data.docx]

**Preoperative Stereotactic Radiotherapy to Prevent Pancreatic Fistula in High-Risk Patients Undergoing Pancreatoduodenectomy (FIBROPANC): A Prospective Multicenter Phase II Single-Arm Trial**

Leonoor V Wismans^1,2*^, Tessa E Hendriks^3,4,5*^, J Annelie Suurmeijer^3,4^, Joost J Nuyttens^6^ Anna M Bruynzeel^3,7^, Martijn P. Intven^8^, Lydi M van Driel^9^, Roel Haen^1,2^, Roeland F. de Wilde^1,2^, Bas Groot Koerkamp^1,2^, Olivier R Busch^3,4^, Jaap Stoker^4,10^, Joanne Verheij^4,11^, Arantza Farina ^4,11^, Onno J. de Boer^4,11^, Michail Doukas^1,12^, Ignace H de Hingh^13^, Daan J Lips^14^, E van der Harst^15^, Geertjan van Tienhoven^3,16#^ Casper H van Eijck^1,2#^, Marc G Besselink^3,4#^ for the Dutch Pancreatic Cancer Group

1 Erasmus MC Cancer Institute, Erasmus MC, University Medical Center Rotterdam

2 Department of Surgery, Erasmus MC, University Medical Center Rotterdam

3 Amsterdam UMC, location University of Amsterdam, Department of Surgery, Amsterdam

4 Cancer Center Amsterdam

5 Department of Surgery, Leiden University Medical Center, Leiden

6 Department of Radiation Oncology, Erasmus University Medical Center, Rotterdam

7 Amsterdam UMC, location Vrije Universiteit, Department of Radiation Oncology, Amsterdam

8 Department of Radiation Oncology, University Medical Center Utrecht, Utrecht

9 Department of Gastroenterology and Hepatology, Erasmus University Medical Center, Rotterdam

10 Amsterdam UMC, location University of Amsterdam, Department of Radiology and Nuclear Medicine, Amsterdam

11 Amsterdam UMC, location University of Amsterdam, Department of Pathology, Amsterdam

12 Department of Pathology, Erasmus University Medical Center, Rotterdam

13 Department of Surgery, Catharina Hospital, Catharina Cancer Institute, Eindhoven

14 Department of Surgery, Medisch Spectrum Twente, Enschede

15 Department of Surgery, Maasstad Hospital, Rotterdam

16 Amsterdam UMC, location University of Amsterdam, Department of Radiation Oncology, Amsterdam

All: the Netherlands

**Corresponding Author:**

Marc G. Besselink, MD, PhD
Department of Surgery, Cancer Center Amsterdam

Amsterdam UMC, University of Amsterdam
De Boelelaan 1117 (ZH-7F), 1081 HV Amsterdam, the Netherlands
Phone: +31 20 4444 00 | Email: [m.g.besselink@amsterdamUMC.nl](mailto:m.g.besselink@amsterdamUMC.nl)

X: MarcBesselink

**Supplementary Materials - Index**

| **Supplementary Figures and Tables** |  |
| --- | --- |
| Figure 1 | *3* |
| Table 1 | *4* |
| Table 2 | *5* |
| Table 3 | *6* |
| Table 4 | *7* |

**Supplementary Figures and Tables**


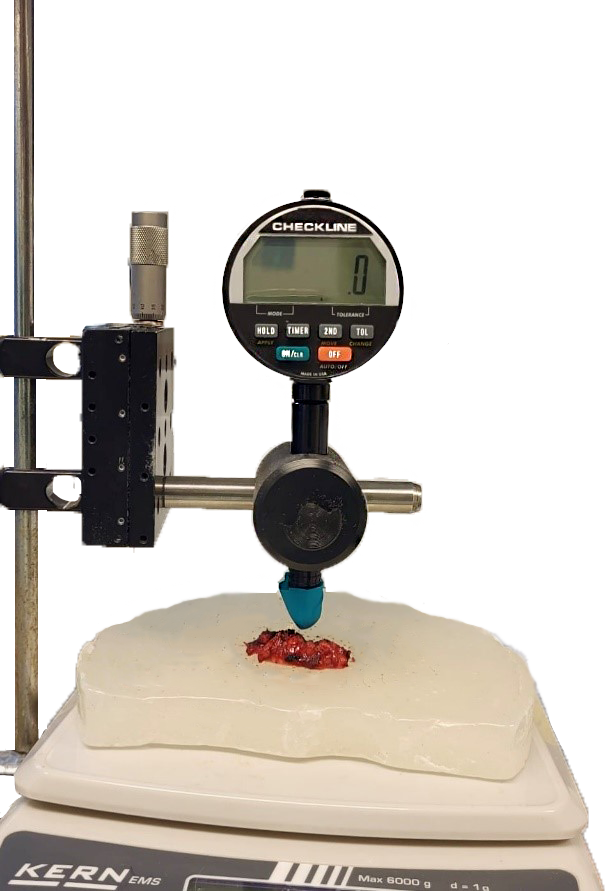


**Supplementary Figure 1**. Durometer set up. The durometer was secured in the operating stand. The sample was positioned under the Durometer and pinned to paraffin to immobilize. The measurement was performed by pressing 500 grams on the sample.

**Supplementary Table 1 -** All grade ≥3 adverse events and relation to study treatment

|  | | | | | | | | | |
| --- | --- | --- | --- | --- | --- | --- | --- | --- | --- |
|  | **Fiducial marker placement** | | | **SBRT** | | | **Unrelated** | | |
|  | **3** | **4** | **5** | **3** | **4** | **5** | **3** | **4** | **5** |
| **Pancreatitis** | **1** | **-** | **-** | **-** | **-** | **-** | **-** | **-** | **-** |
| **Colitis hemicolectomy** | **-** | **-** | **-** | **-** | **-** | **-** | **-** | **1** | **-** |
| **Cholangitis** | **-** | **-** | **-** | **-** | **-** | **-** | **1** |  |  |
| **Fall** | **-** | **-** | **-** | **-** | **-** | **-** | **1** | **-** | **-** |

**Supplementary Table 2.** All grade 2 or lower adverse events and relation to study treatment

|  | **Fiducial marker placement** |  | **SBRT** |  | **Unrelated** |  |
| --- | --- | --- | --- | --- | --- | --- |
|  | Gr. 1 | Gr. 2 | Gr. 1 | Gr. 2 | Gr. 1 | Gr. 2 |
| Delayed systemic treatment (postoperative diagnosis PDAC) | - | - | - | 1 |  |  |
| Fatigue | - | - | 2 | - | - | - |
| Abdominal pain | - | - | 5 | 1 | - | - |
| Nausea | - | - | 3 | - | - | - |
| Vomiting | - | - | 4 | - | - | - |
| Diarrhea | - | - | 2 | - | - | - |
| Constipation | - | - | 1 | - | - | - |
| Cystitis | - | - | - | - | 1 |  |
| Reflux | - | - | 1 | - | - | - |
| ***Total SAE count*** | 0 | 0 | *18* | *2* | *1* | *0* |

**Supplementary Table 3.** Outcomes of firmness (Durometer) and fibrosis (collagen density) assessments between the sample of the resection margin (irradiated) and the uncinate process (non-irradiated).

| **Study participants** | **Number of patients** | **Resection margin** | **Processus uncinatus** | **p-value** |
| --- | --- | --- | --- | --- |
| Durometer assessment , ShoreOO | 33 | 47 [36-57] | 37 [30-41] | <0.001 |
| Collagen density, % sirius red area | 33 | 6.1 % [4.4-9.5] | 4.5 % [2.5-7.4] | 0.0031 |
|  |  |  |  |  |
| **Control patients** | **Number of patients** | **Resection margin** | **Processus uncinatus** | **p-value** |
| Durometer assessment , ShoreOO | 7 | 40 [35-56] | 43 [34-49] | 0.58 |
| Collagen density, % sirius red area | 3 | 12.2 [8.1-28.6] | 12.1 [9.0-16.1] | 0.75 |
| *Median [IQR]* | | | | |

**Supplementary Table 4.** Potential risk factors for POPF in audit controls.

|  | **Audit controls (n = 134)**^1^ |
| --- | --- |
| **Age** | 67 (60, 73) |
| Missing | 1 |
| **BMI** | 25.5 (22.5, 28.9) |
| Missing | 3 |
| **History of Pancreatitis** | 3 (2.5%) |
| Missing | 15 |
| **History of Diabetes** | 17 (16%) |
| Missing | 26 |
| **Diagnosis** |  |
| Cholangiocarcinoma | 37 (28%) |
| Papillary cancer | 25 (19%) |
| Duodenal cancer | 10 (7.5%) |
| Neuroendocrine neoplasm | 15 (11%) |
| IPMN, SPN, MCN | 19 (14%) |
| Other/unknown | 28 (21%) |
| **Duct diameter (cm)** |  |
| 1 | 13 (9.7%) |
| 2 | 58 (43%) |
| 3 | 63 (47%) |
| **POPF** |  |
| No | 87 (66%) |
| Grade B/C | 45 (34%) |
| Missing | 2 |
| ^1^Median (IQR); n (%) | |
